# Rats Bred for Low Aerobic Capacity Become Promptly Fatigued and Have Slow Metabolic Recovery after Stimulated, Maximal Muscle Contractions

**DOI:** 10.1371/journal.pone.0048345

**Published:** 2012-11-20

**Authors:** Sira Torvinen, Mika Silvennoinen, Harri Piitulainen, Johanna Närväinen, Pasi Tuunanen, Olli Gröhn, Lauren G. Koch, Steven L. Britton, Heikki Kainulainen

**Affiliations:** 1 Department of Biology of Physical Activity, Neuromuscular Research Center, University of Jyväskylä, Jyväskylä, Finland; 2 Department of Neurobiology, AI Virtanen Institute for Molecular Sciences, University of Eastern Finland, Joensuu, Finland; 3 Brain Research Unit, Low Temperature Laboratory, School of Science, Aalto University, Espoo, Finland; 4 Department of Anesthesiology, University of Michigan, Ann Arbor, Michigan, United States of America; University of Sydney, Australia

## Abstract

**AIM:**

Muscular fatigue is a complex phenomenon affected by muscle fiber type and several metabolic and ionic changes within myocytes. Mitochondria are the main determinants of muscle oxidative capacity which is also one determinant of muscle fatigability. By measuring the concentrations of intracellular stores of high-energy phosphates it is possible to estimate the energy production efficiency and metabolic recovery of the muscle. Low intrinsic aerobic capacity is known to be associated with reduced mitochondrial function. Whether low intrinsic aerobic capacity also results in slower metabolic recovery of skeletal muscle is not known. Here we studied the influence of intrinsic aerobic capacity on in vivo muscle metabolism during maximal, fatiguing electrical stimulation.

**METHODS:**

Animal subjects were genetically heterogeneous rats selectively bred to differ for non–trained treadmill running endurance, low capacity runners (LCRs) and high capacity runners (HCRs) (n = 15–19). We measured the concentrations of major phosphorus compounds and force parameters in a contracting triceps surae muscle complex using ^31^P-Magnetic resonance spectroscopy (^31^P-MRS) combined with muscle force measurement from repeated isometric twitches.

**RESULTS:**

Our results demonstrated that phosphocreatine re-synthesis after maximal muscle stimulation was significantly slower in LCRs (p<0.05). LCR rats also became promptly fatigued and maintained the intramuscular pH poorly compared to HCRs. Half relaxation time (HRT) of the triceps surae was significantly longer in LCRs throughout the stimulation protocol (p≤0.05) and maximal rate of torque development (MRTD) was significantly lower in LCRs compared to HCRs from 2 min 30 s onwards (p≤0.05).

**CONCLUSION:**

We observed that LCRs are more sensitive to fatigue and have slower metabolic recovery compared to HCRs after maximal muscle contractions. These new findings are associated with reduced running capacity and with previously found lower mitochondrial content, increased body mass and higher complex disease risk of LCRs.

## Introduction

Muscular fatigue is characterized with decline of force production caused by prolonged activation of skeletal muscle. It is known that muscle fiber types, characterized by differences in myosin heavy chain (MHC) composition, vary in force-generating capacity and resistance to fatigue [1]–[4]. Hence muscle fiber type composition is one key factor affecting the sensitivity of muscle to fatigue. At a myocellular level the reduced force can be caused by various metabolic or ionic changes, such as reduced pH, accumulation of inorganic phosphate (Pi) into myocytes, decreased intracellular calcium ion (Ca^2+^) release or reduced sensitivity of the myofilaments to Ca^2+^-ions [5], [6]. These changes in myocellular metabolites during muscle fatigue are often linked to each other. For example reduced pH and accumulation of Pi lead to reduced Ca^2+^ sensitivity and reduced maximum tension. This in turn has an important contribution to the force decline, especially with repeated maximal muscle stimulation [7].

In addition to muscle fiber type composition and myocellular metabolic and ionic changes the capacity to resist fatigue is determined by muscle oxidative capacity [8]–[10]. Muscle oxidative capacity in turn is mainly determined by the amount and quality of myocellular mitochondria. These parameters vary between individuals due to differences in genotype and in the amount of physical activity [11]–[14]. Besides being a major contributor to muscle oxidative capacity, mitochondria have a crucial role in integrating the key metabolic fluxes in the cell. Phosphocreatine (PCr) and creatine (Cr) provide an intracellular, high-energy phosphate buffering system, essential to maintain adenosine-triphosphate (ATP) levels in tissues with high energy demands. Internal stores of high-energy phosphates provide additional ATP at the onset of exercise in skeletal muscle.

With ^31^P-Magnetic resonance spectroscopy (^31^P-MRS) it is possible to measure the concentrations of the major phosphorus compounds in a contracting muscle, *i.e.*, PCr and Pi [15], [16]. With these parameters one can estimate the energy production efficiency of the muscle, such as the speed of PCr degradation and resynthesis, provided that the simultaneous force output is recorded [17]. Until recently, this method required invasive systems to induce muscle contraction electrically, which in turn necessitated the sacrifice of the animal after the experiment. Giannesini and colleagues (2005) designed and constructed a new experimental setup for noninvasive MRS investigation of muscle function in contracting rat triceps surae muscle complex (gastrocnemius, soleus and plantaris) [18]. This maximal intermittent isometric fatigue protocol provides a reliable way to study the development of muscle fatigue of the whole muscle without occlusion of the blood flow. Due to the noninvasive approach, muscle metabolic properties can be followed through an individual's life-span, which enables the evaluation of inherited muscle properties together with effects of environmental factors (*e.g.* exercise training) with aging.

Recent studies reveal that low aerobic capacity is associated with various metabolic and morphological properties in skeletal muscle, which in turn have pronounced effects on whole body level in health and disease [19]–[23]. Since impaired oxidative metabolism underlies several complex diseases, such as type 2 diabetes and cardiovascular disease [24], [25], it is generally thought that the ability to increase oxygen transport and utilization capacity by exercise training stimulus helps to prevent or ameliorate developing complex disease. The level of exercise capacity, however, differs substantially between individuals. The primary assumption is that this heterogeneity is a consequence of variation distributed across a large number of genes for both the intrinsic (untrained) aerobic capacity and that accrued from exercise training. To address the intrinsic component, Koch and Britton developed a contrasting animal model system by selectively breeding rats for low and high endurance treadmill running capacity in the untrained condition [10]. These genetically heterogeneous and widely segregated rat strains, termed low capacity runner (LCR) and high capacity runner (HCR), are well suited divergent models for evaluating the interplay of genetic and environmental factors as determinants of health and disease.

Since intrinsic aerobic capacity has pronounced effects in health, complex disease risk and life expectancy [19], [22], [26] the differences on whole body level of HCR/LCR rats should also be observable in tissue level. In this study the focus is on skeletal muscle. By strict measurement criteria, LCRs fatigue sooner during maximal treadmill running test compared to HCRs [10] but it is not known whether these strains differ in parameters related to maximal muscle stimulation. Therefore, the purpose of this study was to examine the effects of intrinsic aerobic capacity on skeletal muscle metabolism *in vivo* during maximal, fatiguing stimulation in non-trained LCR/HCR rats. We hypothesize that low intrinsic aerobic capacity is associated with fatigue sensitivity, slow metabolic recovery and poor intramuscular pH homeostasis. We also hypothesize, that compared to HCRs, the twitch parameters of LCRs are more typical of fast twitch muscles due to larger relative amount of type 2b muscle fibers [19], [27], [28].

## Materials and Methods

The HCR/LCR contrasting rat model system was produced via two-way artificial selection, starting from a founder population of genetically heterogeneous rats (N:NIH stock) in 1996, as described previously [10]. Endurance running capacity was assessed at the University of Michigan (Ann Arbor, Michigan, USA) with a speed-ramped treadmill running test (15° slope, initial velocity of 10 m min^−1^, increased 1 m min every 2 min) when the rats where 11 weeks of age (model Exer-4; Columbus Instruments, Columbus, OH). Rats were subsequently shipped to University of Jyväskylä when 16 weeks old. For experiments described here, 34 female HCR and LCR rats (nHCR = 19, nLCR = 15) weighing 194–332 g were used. Rats were from the 23rd generation of selection and 8 months of age when studied. Rats were housed 2/cage in an environmentally controlled facility (12/12 h light-dark cycle, 22°C) and received water and standard feed *ad libitum*.

### Ethics Statement

This study was approved by the National Animal Experiment Board, Finland (Permit number ESAVI-2010-07989/Ym-23). The 31P-MRS acquisition protocol was performed under isoflurane anesthesia.

### Animal Preparation

**Figure 1:**
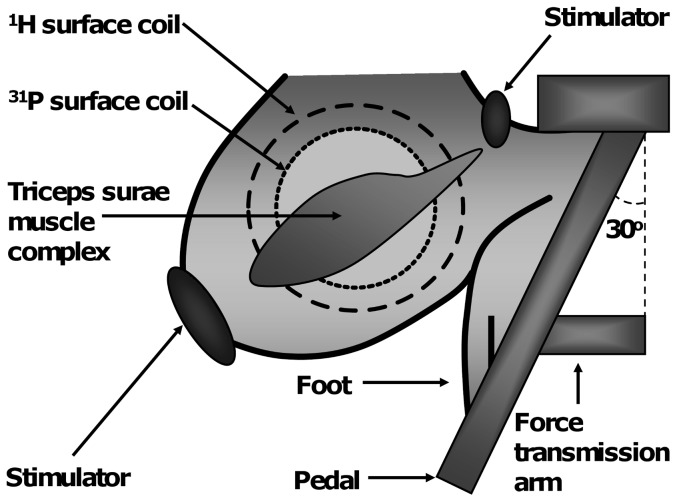
Schematic representation of the setup for measuring rat triceps surae muscle complex function with MR investigation. Triceps surae contractions were induced indirectly via electrical stimulation at the heel and knee levels. Muscle performance was measured with a force transducer which was attached to the pedal. Information about the leg position was acquired with a ^1^H-MR surface coil and muscle metabolic functions were studied with ^31^P-MRS surface coil.

The non-invasive MRS investigation setup was modified from the protocol designed by Giannesini et al. [18]. Rats were deprived of food for 2 h before measurements. Basal body temperature and masswere measured at rest before each investigation. Rats were anesthetized in an induction chamber with 4% isoflurane mixed in 30% O_2_ and 70% N_2_O. The right lower hindlimb was shaved and conductive electrolyte gel was applied at the heel and knee levels to optimize electrical stimulation of triceps surae muscle complex. Each rat was placed in a home-built cradle designed for non-invasive functional investigation of the right triceps surae. The cradle had a built-in strain-gauge sensor (HBM, 1-LY41-6/1000, Darmstadt, Germany) and two transcutaneous electrodes to elicit and measure maximal twitch responses under isometric conditions. The foot was positioned on the pedal and the lower hind limb was centered on a flat radio frequency (RF) coil used for MRS measurement (Fig. 1). The pedal was held constant at a 30 degree angle. Supramaximal square-wave pulses (1-ms duration, current range: 10–19 mA, altered to maximize twitch force) were delivered transcutaneously with a constant current stimulator (Digitimer Stimulator DS7, Digitimer Ltd., Hertfordshire, U.K.) to obtain the maximal isometric twitch responses. Throughout the experiment, anesthesia was maintained by a gas inhalation through a facemask continuously supplied with 1.2–1.75% isoflurane in 30% O_2_ and 70% N_2_O. Corneas were protected from drying by application of ophthalmic cream (Viscotears, Novartis Pharmaceuticals, U.K.). The facemask was connected to open-circuit gas anesthesia equipment. During anesthesia, animal body temperature was maintained with a heated pad.

### 31P-MRS Acquisition and Data Processing

Investigations were performed in a 4.7 T horizontal magnet (Magnex Scientific, Abdington, U.K.). A flat surface transmit/receive RF coil (26-mm-diameter ^1^H- and 20-mm-diameter ^31^P-coil) was used. The triceps surae muscle complex was carefully placed in the center of the coil combination, and the volume on coil was shimmed using ^1^H nuclear magnetic resonance (NMR) signal followed by pilot images with gradient echo pulse sequence to ensure that the position of the muscle was correct. ^31^P-MR spectra from the triceps surae were continuously acquired with pulse-acquire technique using 130 µs long, 90 degree block pulse for excitation, 1.855 s time to repeat, 4 kHz bandwidth covered with 2000 points. Prior to stimulation, a baseline spectrum was collected (256 averages, 8 min) and during the stimulation and recovery, a total of 15 spectra were acquired, resulting in temporal resolution of 56 seconds. Areas of PCr and Pi were obtained by a time-domain fitting routine using the AMARES-MRUI Fortran code [29]. PCr and Pi values were normalized to their initial values. Intramuscular pH was calculated from the chemical shift difference between Pi and PCr [30]. The recovery of PCr level was compared by adjusting a linear trend line between time points 7–9 min and comparing the individual slope values. The chosen time-interval showed rapid linear increase in the analyzed variable.

### Stimulation Protocol and Signal Analysis

The stimulation protocol consisted of baseline (8 min), stimulation (6 min) and recovery (8 min) measurements. Repeated isometric muscle twitches were elicited through electrical stimulation of triceps surae muscle complex at a frequency of 3.33 Hz (in total 1200 stimuli). Force signal from the strain-gauge sensor was amplified, converted to digital signals by a 32-bit analog to digital converter (Power 1401, CED Ltd., Cambridge, U.K.), and processed using dedicated software (Signal software, CED Ltd.). From each individual twitch response, 1) maximal force, 2) maximal rate of torque development (MRTD) and 3) half-relaxation time (HRT) were analyzed throughout the stimulation period. The twitch properties were then averaged over 15 second epochs (in total 24 averages of 50 stimuli). In addition, twitch force and MRTD values were normalized with their maximum value during the stimulation to compute relative rate of reduction (% of maximum s^−1^) during the time-course of transcutaneous electrical stimulation. A linear trend line was adjusted over 100 twitch responses in individual time-intervals that showed rapid linear reduction in the analyzed variables.

### Statistical Analyses

All values are expressed as mean ± standard error of the mean (SEM). Statistical analyses for all variables were carried out using SPSS for Windows 13.0 statistical software (SPSS Inc., Chicago, IL, USA). The Shapiro-Wilk test was used to investigate within group normality for a given parameter of interest. Levene's test was conducted to assess the homogeneity of the variance assumption. When assumptions were met independent-samples t-test was used. A statistical comparison of body mass and mean distance run to exhaustion was made using independent-samples t-test. The recovery of PCr level was compared with independent-samples t-test by comparing the individual slope values. Since the normality or equality of variance assumptions were not met, statistical comparisons of other studied parameters between LCR and HCR groups were made using Mann-Whitney test by comparing LCRs and HCRs at every time point. The twitch property values from the first last epochs (Table 1) were also compared using Mann-Whitney test. The rate of reduction in relative twitch force and MRTD (Table 2) were analyzed using Mann-Whitney test by comparing the individual slope values. P-values less than 0.05 were considered statistically significant.

**Table 1:** Comparison of initial and end values of the twitch properties.

Parameter	Epoch	LCR	HCR	P
Twitch force	First	2.87±0.25	2.51±0.13	0.206
(N)	Last	1.01±0.11	1.29±0.10	<0.05
MRTD	First	184±17	167±10	0.477
(N s-1)	Last	75±8	98±9	<0.01
HRT	First	16.5±0.4	14.1±0.3	<0.001
(ms)	Last	15.2±0.4	13.5±0.5	<0.01

Initial values of twitch force, MRTD and HRT from First epoch (0–15 s) and from Last epoch (345–360 s) during the electrical stimulation. Values are expressed as mean ± SEM.

**Table 2:** Rate of change in twitch properties.

Parameter	LCR	HCR	P
Slope	−0.19±0.02	−0.13±0.06	<0.05
(Force % of max min-1)			
Slope	−0.17±0.02	−0.12±0.01	<0.05
(MRTD % of max min-1)			

The rates of change in relative (% of maximal) twitch force and MRTD in individual time-intervals (100 twitches) during the electrical stimulation. Values are expressed as mean ± SEM.

## Results

### Background Data

LCR rats were 26% heavier compared to HCRs, the mean body mass of LCRs was 289±7 g and for HCRs 229±5 g (p<0.001). The mean distance run at exhaustion was 305±4 m for LCRs *versus* 2093±25 m for HCRs (p<0.001), representing a 586% difference in running distance.

### ^31^P-MRS

#### PCr

**Figure 2:**
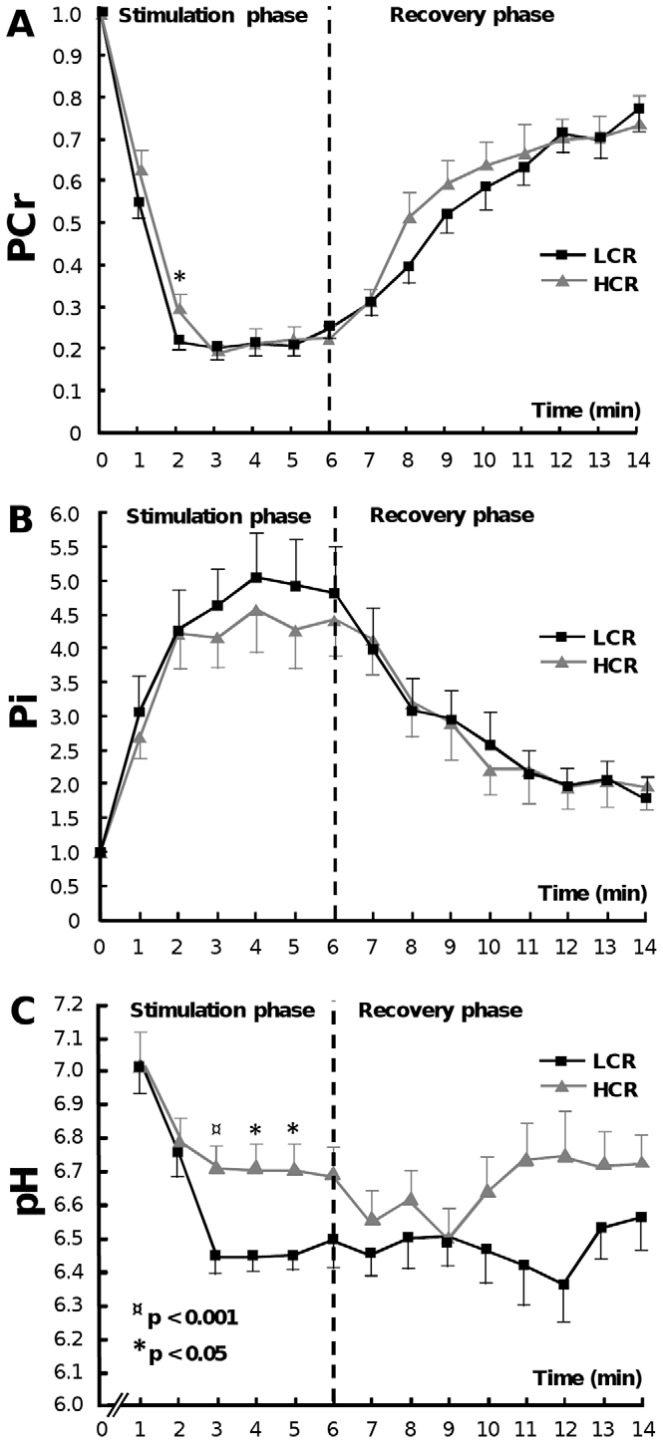
PCr, Pi and pH levels during ^31^P-MRS acquisition protocol. PCr (A), Pi (B) and pH (C) levels in triceps surae muscle complex during stimulation (6 min) and recovery (8 min) measurements. PCr resynthesis was significantly slower in LCRs compared to HCRs (p<0.05) when comparing the individual slope values. LCRs also had significantly lower PCr level in time point 2 min (p<0.05). There were no statistical differences between HCR and LCR groups in Pi levels. Intramuscular pH was lower in LCRs throughout the protocol except for the time points 1 and 9 min. Values are expressed as mean ± SEM.

^31^P-MRS results demonstrated that PCr resynthesis after stimulation was significantly slower in LCRs when comparing the slope values of linear trend lines adjusted between time points 7–9 min (p<0.05) (Fig. 2A). In both rat strains, the decrease of PCr from the resting level (t = 0) started right after stimulation. PCr level also began to increase in both strains immediately after the stimulation protocol was over (t = 6). LCR rats had significantly lower PCr value in time point 2 min of stimulation compared to HCR rats.

#### Pi

Pi levels increased similarly in both rat strains during stimulation period and reached the highest level after 4 minutes of stimulation (Fig. 2B). During recovery period Pi decreased and almost reached back to the starting level. There were no significant differences in the levels of Pi between the groups.

#### pH

LCRs maintained the intramuscular pH more poorly compared to HCRs (Fig. 2C). LCRs had significantly lower pH values between stimulation periods 3–5 min and the levels remained lower than the corresponding pH levels of HCRs during the whole protocol, except for the time points 0 and 9 min. At the beginning of the stimulation protocol both rat strains had an equivalent pH value of 7.00. At end of the test period, HCRs had on average a pH of 6.78, whereas LCRs had more reduced pH of 6.55.

### Twitch Properties

#### Initial and End Values of the Twitch Properties

**Figure 3:**
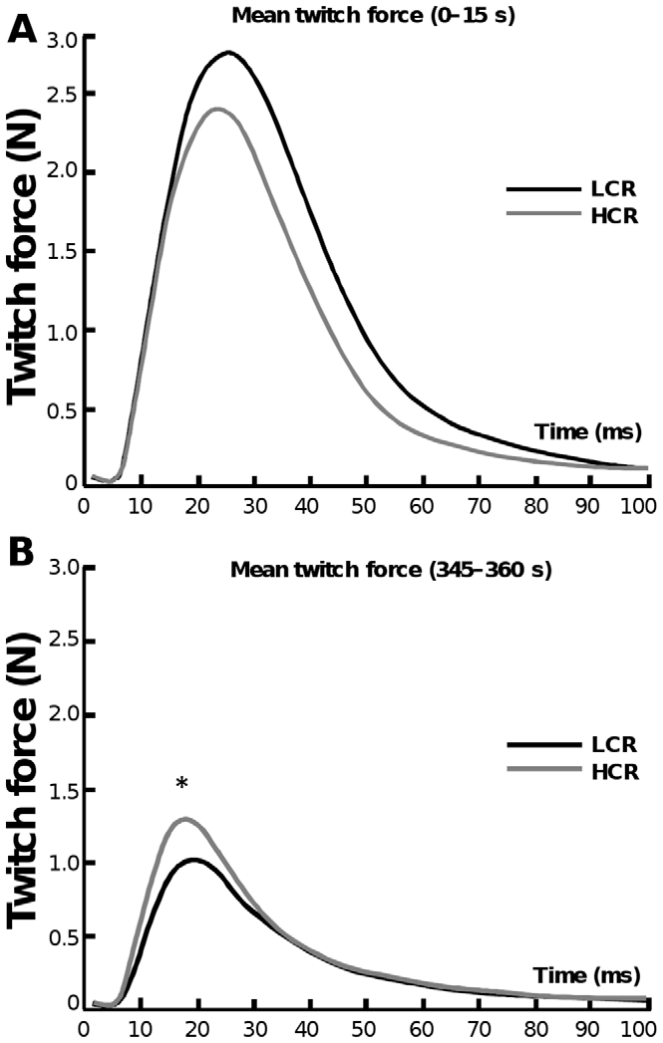
Initial and end values of the twitch properties. The mean twitch force curves from initial epoch (0–15 s) (A) and from last epoch (345–360 s) (B) during electrical stimulation. The corresponding twitch property values are presented in Table 1. LCRs seemed to have slightly higher initial maximal twitch force and MRTD (n.s.). At the end of the stimulation protocol LCRs showed significantly lower maximal twitch force and MRTD compared to LCRs (p<0.05). LCRs had significantly higher HRT values both in first and last epoch (p<0.01) than HCRs.

Mean twitch force curves from the first epoch (0–15 s) and from the last epoch (345–360 s) are presented in figures 3A and 3B. The corresponding twitch property values are presented in Table 1. During the first epoch, when the effect of fatigue is minimal, LCRs seemed to show higher initial maximal twitch force, although this was not statistically significant. However, the initial MRDT did not differ between LCRs and HCRs. As the effect of fatigue LCRs showed lower maximal twitch force and MRTD compared to HCRs during the last epoch (p<0.05). LCRs also had significantly lower MRTD in the last epoch (p<0.01) and higher HRT values both in first and last epoch (p<0.01) compared to HCRs.

#### Twitch Properties during Electrical Stimulation

**Figure 4:**
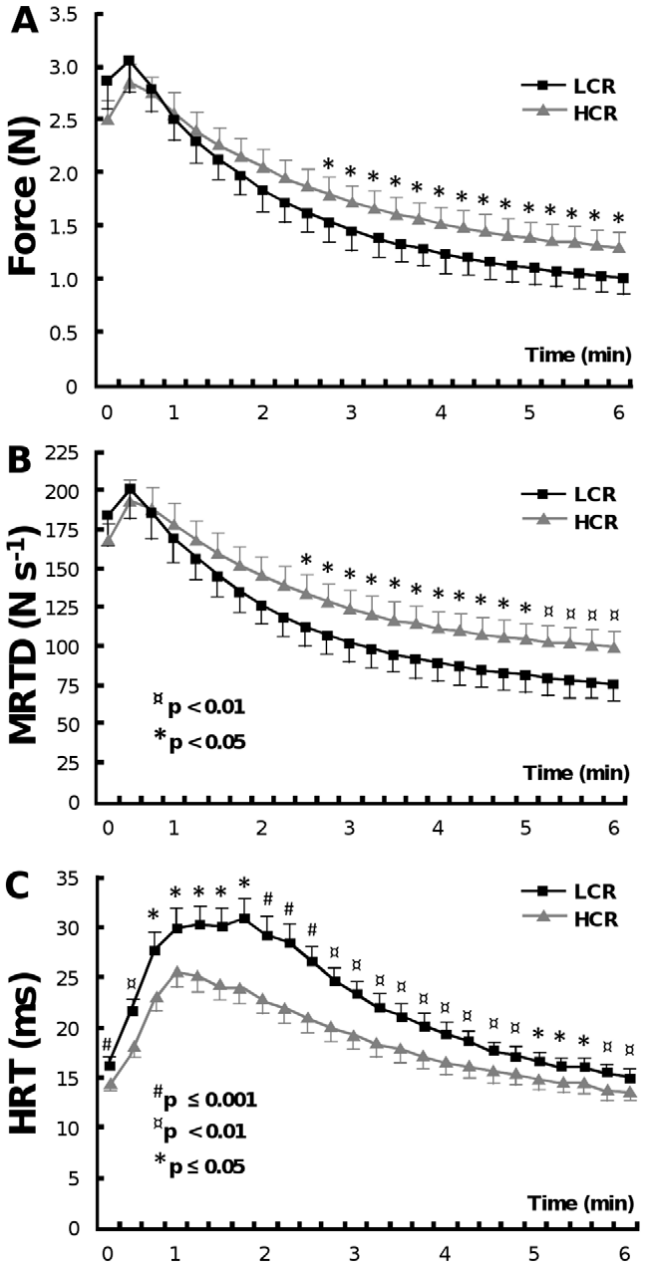
Twitch properties during electrical stimulation. Force (A), MRTD (B) and HRT (C) measured simultaneously with MRS acquisition during 6 min stimulation protocol. From 2 min 45 s onwards LCRs had significantly lower twitch force compared to HCRs (p≤0.05). The MRTD values were significantly lower in LCRs compared to HCRs from 2 min 30 s onwards (p≤0.05). LCRs had higher HRT than HCRs throughout the stimulation protocol (p≤0.05). Values are expressed as mean ± SEM.

LCRs tended to show higher maximal twitch force than HCRs during the first epochs (0–30 s), although this trend was not significant. Later during the stimulation, LCRs showed a significantly lower twitch force from 2 min 45 s onwards compared to HCRs (p≤0.05) (Fig. 4A). Similarly, LCRs had tendency for higher MRTD (N s^−1^) in the beginning of the stimulation protocol than HCRs (Fig. 4B), but this difference did not reach significance. However, the MRTD was significantly lower in LCRs compared to HCRs from 2 min 30 s onwards (p≤0.05). The half relaxation time (Fig. 4C) of the triceps surae was significantly longer in LCRs throughout the stimulation protocol (p≤0.05).

#### Rate of Change in Twitch Properties

The rate of reduction in the normalized twitch force and MRTD during transcutaneous electrical stimulation occurred more rapidly in LCRs compared to HCRs (p<0.05) when the slope of individual twitch responses were compared (Table 2).

## Discussion

In the present study we examined noninvasively the characteristics of triceps surae muscle complex metabolism in two genetically heterogeneous rat strains (HCR/LCR) that widely differ for intrinsic (i.e. non-trained) aerobic capacity. Consistent with a lower maximal treadmill running capacity, we found that the skeletal muscle in LCRs become promptly fatigued during maximal muscle stimulation *in vivo*. LCRs have also slower metabolic recovery after continued contractions compared to HCRs.

We hypothesized, that LCRs would have slow metabolic recovery, which is one sign of poor mitochondrial function and/or capacity [30], [31]. In previous studies we have shown, that LCRs have poor capillarization and less subsarcolemmal and intermyofibrillar mitochondria in skeletal muscle compared to HCRs [19]. Rivas *et al* in turn found that the most significant differences in mitochondrial content between these rat strains were in white muscle type [32]. It is likely that the slower PCr recovery of LCRs is partly due to lower mitochondrial content of the muscle compared to HCRs. Diminished oxidative capacity of the mitochondria may also play a role. Indeed, it has been shown that despite similar mitochondrial density, LCRs have reduced mitochondrial respiratory capacity in skeletal muscle compared to HCRs [33]. This is consistent with the finding, that skeletal muscle from obese subjects or from type 2 diabetic patients show reduced mitochondrial oxidative capacity [20], [23], [25], [34].

A novel finding from the present study was that LCRs become promptly fatigued during maximal isometric muscle stimulation (Fig. 4 and Table 2), not only during an aerobic running test. In the present study we used protocol with intermittent isometric twitches used by Giannesini et al [18]. Isometric muscle stimulation method enabled us to hold the muscle still during the whole stimulation period, which is vital to ^31^P-MRS investigation when using surface coils. Another advantage of the isometric fatigue protocol is the constant muscle length that ensures measured force parameters are not affected by change in the muscle length. The non-invasive muscle stimulation method has been proven to stimulate solely the triceps surae muscle complex and not the antagonist muscles. In fact, Giannesini et al (2005) found in their study, using T_2_-weighted MR images, that the transcutaneous stimulation system activates specifically the gastrocnemius muscle of triceps surae [18]. Since our stimulation system is similar to that used in Giannesini's studies we assume, that also our transcutaneous stimulation mainly stimulates gastrocnemius muscle.

Muscle fibers vary in force-generating capacity, contraction speed and resistance to fatigue [2], [3]. Previous analysis of MHC composition revealed that LCRs express more 2b MHC isoform compared to HCRs, where 2a/x MHC was more pronounced [19]. MHC types define the muscle fiber type, which in turn belong to motor units having distinct functional properties: type 2a fibers correspond to fast-contracting fatigue-resistant motor units and type 2b fibers to fast-contracting fatigue-sensitive motor units [4]. Howlett *et al* also showed that LCRs have significantly higher activation of the glycolytic enzyme phosphofructokinase [35]. Our results from force measurement support these findings. During the maximal stimulation LCRs fatigued at a faster rate, whereas HCRs demonstrated fatigue resistance (Table 2). MRTD is a muscle twitch parameter that illustrates how fast the maximal torque level is reached. Muscles that consist mainly of fast-twitch fibers tend to have higher MRTD values. Although LCRs start with a higher twitch force and MRTD at the beginning of the stimulation (0–15 s) the opposite occurs upon reaching the end of the stimulation protocol (345–360 s) (Fig. 4). This supports our hypothesis, that LCRs have greater reliance on fast-contracting and fatiguing muscle fibers compared to HCRs.

It has been shown that HCR and LCR rats have similar resting muscle phosphocreatine, glycogen and ATP contents [35]. LCRs, however, have diminished sensitivity for Cr-induced stimulation of submaximally ADP-stimulated respiration in skeletal muscle compared HCRs [33]. This tendency was also true in the case of non-ADP-stimulated and maximally ADP-stimulated respiration. A diminished sensitivity in skeletal muscle may contribute to the performance differences between LCR and HCR rats, as a consequence of an increased insensitivity of the mitochondrion, and thus the whole muscle, to ADP during exercise. The work of Walsh *et al* demonstrated that HCRs maintain a higher degree of functional coupling between creatine kinase and adenine nucleotide translocase compared to the LCRs in slow-twitch muscle type [33]. Whether these qualitative changes in mitochondrial function are related to inherent muscle oxidative capacity or are directly in response to exercise capacity remains unclear.

In skeletal muscle, the fatiguing stimulation resulted in depletion of PCr, accumulation of Pi, and a decrease in pH. As we hypothesized, LCRs had poor intramuscular pH homeostasis compared to HCRs. The larger contractile-mediated decrease in pH in the triceps surae of LCRs was likely related to the larger glycolytic capacity in fast-twitch muscle fibers (type 2b) of LCRs when compared to HCRs [19], [36], [37]. More oxidative muscle fibers (type 2a/x) in turn are able to exploit hydrogen ions in the reformation of pyruvic acid and thus maintain a more optimal pH for enzymatic activity longer. In addition to the reduced pH, accumulation of Pi into myocytes has been proposed to play a key role in fatigue development, defined as a decline in force production and slowing of relaxation during prolonged muscle activity [1], [5], [38]. Giannesini *et al* [39] tested five different fatiguing muscle stimulation protocols with the outcome that Pi and its diprotonated form (H_2_PO_4_^−)^ affect force production only at the end of the stimulation period. This suggested an indirect, time-dependent effect of these parameters on force production, which might be mediated by an alteration of Ca^2+^ fluxes throughout the sarcoplasmic reticulum (SR) [39]. In fact, it has been shown, that the decline in Ca^2+^ release from SR occurs later in muscle fibers with high capacity for oxidative metabolism [40], [41]. Thus, HCRs may have more efficient hydrogen ion buffering and delayed decline in Ca^2+^ release due to higher oxidative capacity in skeletal muscle [19], [32], which might help resist fatigue.

We hypothesized, that LCRs might have higher maximal twitch force (N) compared to HCRs due to bigger muscle size and larger relative amount of 2b muscle fibers with better force-generating capacity compared to 2a fibers of HCRs [19], [27], [28]. No significant difference, however, was observed in maximal twitch force between the groups in the beginning of the stimulation protocol. Interestingly, LCRs had significantly longer half-relaxation times compared to HCRs throughout the study protocol (Fig. 4C). This suggests that LCRs have relatively more slow-twitch (type 1) muscle fibers than HCRs. This result is inconsistent with the observation that, in gastrocnemius muscle, LCRs have slightly less oxidative type fibers compared to HCRs [1]. The differences of twitch time parameters of LCR and HCR rats have several possible explanations. The effect of body and muscle size might be again of importance. Bigger muscles and perhaps longer muscle fibers in LCRs could explain the longer relaxation time [42]. Fatigue, due to either Pi, H_2_PO_4_^−^ or Ca^2+^ fluxes, can also increase the half- relaxation time [17].

The possible link between muscle oxidative capacity and metabolic diseases is an interesting topic. We have already shown that low aerobic capacity is related to higher complex disease risk [19]. Our present results suggest that muscle oxidative capacity, muscle fatigue and complex disease risk are connected to each other. Nevertheless, what is the cause and what is the consequence remains unclear. In addition to the study of complex metabolic diseases, there is a growing interest in understanding the connection between aerobic capacity and exercise training in as it relates to longevity. Several large-scale clinical studies have shown that low exercise capacity is the strongest predictor of all-cause mortality [43]–[45]. It has been demonstrated, that in addition to a variety of biochemical and physiological differences, LCRs have significantly shorter lifespan compared to HCRs [26]. Aging is also associated with a decline in the rate of force development, especially during fast contractions [46], [47]. Studies in humans have revealed that HRT increases and the MRTD declines in response to aging [30], [48]. Our present study revealed that, in the non-trained state, rats with low aerobic capacity became promptly fatigued during maximal muscle stimulation and had slow metabolic recovery after continued contractions in triceps surae muscle complex. These results supported the previous findings of substantial differences in skeletal muscle properties between these rat strains [19], [32], [33], [35]. It will be of interest for future studies to determine if exercise training can retrieve the negative contractile activity of the LCR as a function of age and the noninvasive nature of the ^31^P-MRS measures of contractile function as reported here will be of substantial experimental value for studying the complexities of aging.
